# Liraglutide Attenuates Disease Severity in Experimental Autoimmune Encephalomyelitis by Modulating Splenic T Helper Cell Subsets

**DOI:** 10.1002/brb3.71074

**Published:** 2025-11-17

**Authors:** Shuang Song, Bin Li, Ruoyi Guo, Yi Zhou, Yumei Xue, Li Guo

**Affiliations:** ^1^ Department of Neurology The Second Hospital of Hebei Medical University Shijiazhuang Hebei China; ^2^ Key Neurological Laboratory of Hebei Province Shijiazhuang Hebei China; ^3^ Key Laboratory of Clinical Neurology, Ministry of Education Hebei Medical University Shijiazhuang Hebei China; ^4^ Department of Neurology Shijiazhuang People's Hospital Shijiazhuang China

**Keywords:** experimental autoimmune encephalomyelitis, liraglutide, splenocyte

## Abstract

**Objective:**

To investigate the therapeutic effects of liraglutide, a glucagon‐like peptide‐1 receptor agonist, on clinical progression and splenic T‐cell subsets in experimental autoimmune encephalomyelitis (EAE), a murine model of multiple sclerosis.

**Methods:**

EAE was induced in C57BL/6 mice via myelin oligodendrocyte glycoprotein immunization. The mice were divided into three groups: healthy controls (n = 8), EAE (n = 10), and EAE + liraglutide (n = 10). The EAE + liraglutide treatment group received subcutaneous liraglutide (10 µg/kg every other day) starting at 8 days postimmunization. Body weight and disease score were monitored for 30 days, and part of the animals (n = 4 for each group) were sacrificed 20 days postimmunization for flow cytometry of splenocytes, and splenic T helper 1 (Th1) and regulatory T (Treg) cell proportions were analyzed.

**Results:**

Liraglutide significantly reduced maximum clinical scores (EAE 2.50 ± 0.41 versus EAE + liraglutide 1.75 ± 0.59, *p* = 0.005), but no significant differences in body weight were observed between the EAE and EAE + liraglutide groups. EAE induction decreased the proportion of splenic Treg cells (6.38% ± 0.72% versus control 10.55% ± 0.87%, *p* = 0.013), with liraglutide treatment showing no significant effect on Treg proportion relative to EAE alone (7.53% ± 1.54% versus 6.38% ± 0.72%, *p* = 0.975). However, the proportion of Th1 cells significantly increased after EAE induction (11.95% ± 1.58% versus control 6.05% ± 3.23%, *p* = 0.025), which was reduced by liraglutide (5.94% ± 2.53% versus EAE, *p* = 0.025).

**Conclusions:**

The present preliminary findings demonstrate that liraglutide alleviates EAE severity, probably through peripheral immunomodulatory effects of splenic T helper cells.

## Graphical abstract

The glucagon‐like peptide‐1 receptor agonist (GLP‐1RA) liraglutide alleviates disease severity of experimental autoimmune encephalomyelitis (EAE) mice by reducing splenic T helper 1 cell expansion. These findings suggest GLP‐1RA might exert disease‐modifying effects in the EAE model through peripheral immunomodulatory effects of splenic T helper cells.

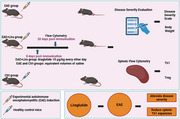

## 1 Introduction

Multiple sclerosis (MS) is a chronic inflammatory demyelinating disorder of the central nervous system (CNS), predominantly affecting young adults with a mean symptom onset age of 30 years (range 20–40 years). While classically considered a disease of early adulthood, emerging epidemiological data reveal a concerning rise in incidence among individuals over 50 years of age (Portaccio et al. 2024). This condition imposes substantial socioeconomic burdens, with a prevalence ranging from 22 to 342 cases per 100,000 population in regions such as North America and Europe (Goodin 2025). Despite advances in disease‐modifying therapies, including monoclonal antibodies, interferon‐β, sphingosine 1‐phosphate receptor modulators, dihydroorotate dehydrogenase inhibitors, glatiramer acetate, and other immunosuppressors (Cohen 2025), therapeutic outcomes remain suboptimal. The global disability‐adjusted life years attributable to MS surged by 66% between 1990 and 2016 (GBD 2016 Neurology Collaborators 2019), underscoring the urgent need for novel therapeutic strategies targeting alternative pathways.

The experimental autoimmune encephalomyelitis (EAE) model remains pivotal for MS research, recapitulating key neuropathological features, including CNS demyelination, inflammatory infiltration, and T cell dysregulation (Capper et al. 2025). Immunization with myelin oligodendrocyte glycoprotein (MOG) induces progressive paralysis in mice, accompanied by T cell imbalance in both lymphoid organs and neural tissues (Azizi et al. 2024, Jia et al. 2022, Chen et al. 2023). This immunophenotypic perturbation mirrors the pathogenic T cell shifts observed in human MS, establishing EAE as a valuable platform for investigating peripheral immune mechanisms.

Liraglutide, a long‐acting glucagon‐like peptide‐1 receptor agonist (GLP‐1RA) with dipeptidyl peptidase‐4 (DPP‐4) resistance, has demonstrated extraglycemic immunomodulatory potential (Diz‐Chaves et al. 2024). Observational studies suggest that GLP‐1RAs may confer vascular risk reduction and weight management benefits in MS patients without significant safety concerns (Udawatta et al. 2025, Balshi et al. 2025, Hardonova et al. 2023). Notably, pharmacovigilance data from the FDA Adverse Event Reporting System indicate an inverse association between liraglutide use and MS (Shirani et al. 2024). Overweight and diabetes may act as both comorbidities and risk factors for MS, thereby underscoring the therapeutic potential of GLP‐1RAs in MS management (Yuan et al. 2021). Preclinical studies further support GLP‐1RA‐mediated neuroprotection and anti‐inflammatory effects in both EAE and cuprizone‐induced demyelination models (Diz‐Chaves et al. 2024, Gharagozloo et al. 2023, Sadek et al. 2023, Ammar et al. 2022, Song et al. 2022, Gharagozloo et al. 2021, Kaye et al. 2024, Chiou et al. 2019). However, critical knowledge gaps persist regarding liraglutide's capacity to directly modulate peripheral immune cell populations in lymphoid organs—a potential mechanism that could synergize with its CNS‐targeted actions.

In this study, we aimed to preliminarily evaluate the therapeutic efficacy of liraglutide in MOG‐induced EAE mice, with dual focus on: (1) clinical outcomes of disease progression and (2) peripheral immunomodulatory effects on splenocytes. Our study elucidates the peripheral effects and mechanisms of liraglutide in MS pathogenesis, providing critical preclinical evidence to support the therapeutic potential of GLP‐1RAs in this disease.

## 2 Materials and Methods

### 2.1 Experimental Animals

Female C57BL/6 mice (8–10 weeks old, 18‐22 g; Beijing Vital River Laboratory Animal Technology Co., Ltd.) were housed under standardized conditions (24°C ± 2°C, 12 h light/dark cycle) with ad libitum access to food and water. The mice were randomly allocated into three groups: the healthy control group (Ctrl group, n = 8), the EAE model group (EAE group, n = 10), and the EAE + liraglutide intervention group (EAE + Lira group, n = 10). Starting at 8 days postimmunization, the treatment group received subcutaneous liraglutide (10 µg/kg every other day) (Song et al. 2022), while EAE controls and healthy controls were administered equivalent volumes of saline. Clinical evaluations (disease scores and body weights) were performed daily. All procedures complied with institutional ethical guidelines (Approval No. 2021‐AE043, Second Hospital of Hebei Medical University).

### 2.2 EAE Induction and Disease Monitoring

EAE was induced in mice via subcutaneous injection of 0.1 mL emulsion containing MOG_35‐55_ peptide (250 µg; Nanjing Peptide Biotech) emulsified in Complete Freund's Adjuvant (CFA; Sigma) with 4 mg/mL *Mycobacterium tuberculosis* H37Ra (Difco) at four paraspinal regions. Pertussis toxin (PTX, 500 ng; List Biological) was administered intraperitoneally on days 0 and 2 postimmunization. Body weight and neurological deficit assessments were performed and recorded daily throughout the observation period until day 30, with investigators blinded to group allocation. Disease severity was scored on a 0–5 scale: 0 (no symptoms), 1 (tail paralysis), 2 (hindlimb paresis), 3 (paraplegia), 4 (forelimb involvement), and 5 (moribund/death), permitting ±0.5 scores for intermediate presentations. (Song et al. 2022) The maximum (max) disease score refers to the highest clinical severity score recorded for each individual mouse during the entire observation period.

### 2.3 Animal Sacrifice and Tissue Preparation

The animals for flow cytometric analysis were sacrificed 20 days postimmunization (when the disease score typically peaks based on previously reported experience (Song et al. 2022)) by quickly dislocating the neck. Subsequently, for the isolation of splenocytes, the fresh spleens were carefully excised and swiftly transferred on ice.

### 2.4 Flow Cytometric Analysis of Splenocytes

Flow cytometric analysis of splenocytes was performed according to previously reported protocols (Guo et al. 2021, Wang et al. 2016). Fresh spleens were carefully homogenized in Roswell Park Memorial Institute 1640 (RPMI 1640) medium supplemented with 10% fetal bovine serum (AusgeneX, Molendinar, Australia) and 1% Penicillin‐Streptomycin Liquid (P1400, Solarbio, Shanghai, China), and filtered through sieves to obtain splenocyte suspensions.

For regulatory T (Treg) cell detection, the cell suspension was directly stained with APC‐labeled anti‐CD25 (AM02505, Multisciences, Hangzhou, China) and FITC‐labeled anti‐CD4 (AM00401, Multisciences, Hangzhou, China) without culture and stimulation process, then fixed and permeabilized by fixation/permeabilization buffer set (88‐17000‐210, eBioscience, San Diego, USA), and subsequently stained with PE‐labeled anti‐forkhead box P3 (Foxp3, A18690, Invitrogen, San Diego, USA) to visualize intracellular representative cytokines in Treg cells.

For T helper 1 (Th1) cell detection, the cell suspension was cultured in 24‐well plates for 24 h before staining (9 h in RPMI 1640 for environmental adaptation and subsequently 15 h in the medium supplemented with 10 µg/ml of MOG_35–55_, phorbol‐12‐myristate‐13‐acetate and ionomycin mixture (CS1001, Multisciences, Hangzhou, China), and Brefeldin A and monensin mixture (CS1002, Multisciences, Hangzhou, China) for stimulation). Then, the cell suspension was stained with FITC‐labeled anti‐CD4, then fixed and permeabilized, and stained with PE‐labeled anti‐interferon‐γ (IFNγ, 12‐1191‐82, eBioscience, San Diego, USA) to visualize intracellular representative cytokines in Th1 cells. Finally, the cells were distinguished and sorted via a FACS‐Calibur flow cytometer (BD Biosciences, San Diego, USA).

### 2.5 Statistical Analysis

Statistical Package for the Social Sciences 21.0 was used to analyze the data. Specifically, the Kruskal‐Wallis test, or analysis of variance test was employed to compare the proportions of T helper cells among different groups, and for comparisons of max disease scores, the Mann‐Whitney test was utilized. In addition, the two‐way Analysis of Variance was adopted to compare the repeated measured data, namely body weight and disease score between groups. A *p*‐value less than 0.05 was regarded as statistically significant.

## 3 Results

### 3.1 Liraglutide Attenuated Disease Scores in EAE Mice

**FIGURE 1:**
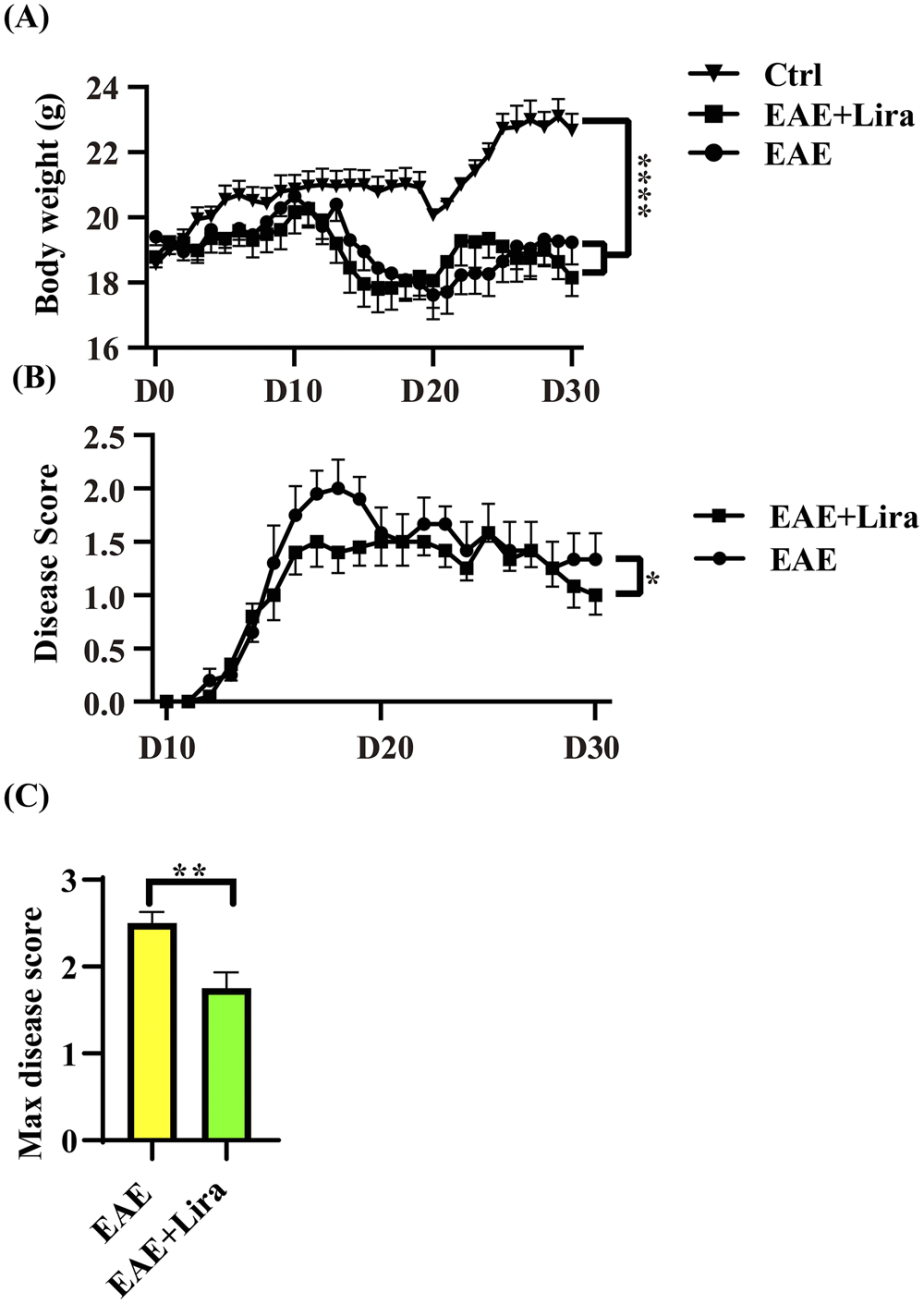
Bodyweight monitor and clinical score of Ctrl, EAE, and EAE + Lira mice. Data were shown with a format of mean ± SEM. * represents *p* < 0.05, ** represents *p* < 0.01, **** represents *p* < 0.0001, and ns represents no significance. (A) Mice body weight was monitored daily after immunization (n = 8 for Ctrl group and n = 10 for the other 2 groups). (B) Disease score between the EAE group and the EAE + Lira group was monitored daily after immunization (n = 10 for each group). The disease score was rated with a five‐point scale: 0, no clinical signs; 1, paralyzed tail; 2, paresis; 3, paraplegia; 4, paraplegia with forelimb weakness or paralysis; 5, moribund or dead. A ±0.5 score was allowed if mice were judged between 2 grades. (C) The max disease scores of mice between the EAE group and the EAE + Lira group were compared (n = 10 for each group).

In the present study, EAE was successfully induced in C57BL/6 mice. In total, 20/20 of the EAE mice (in the EAE and EAE + Lira groups) displayed signs of morbidity, whereas 8/8 healthy control mice did not display any signs of paralysis. Compared with the healthy controls, the mice in the EAE+Lira and EAE groups experienced significant weight loss (*p* < 0.0001), but there was no significant difference between the EAE and EAE+Lira groups (Figure 1A, *p* = 0.118). The disease score was significantly attenuated by liraglutide intervention (Figure 1B, *p* = 0.015), and after liraglutide administration, the maximum disease score was reduced from 2.50 ± 0.41 to 1.75 ± 0.59 (Figure 1C, *p* = 0.005).

### 3.2 Liraglutide Significantly Reduced the Th1 Proportion of Splenocytes

**FIGURE 2:**
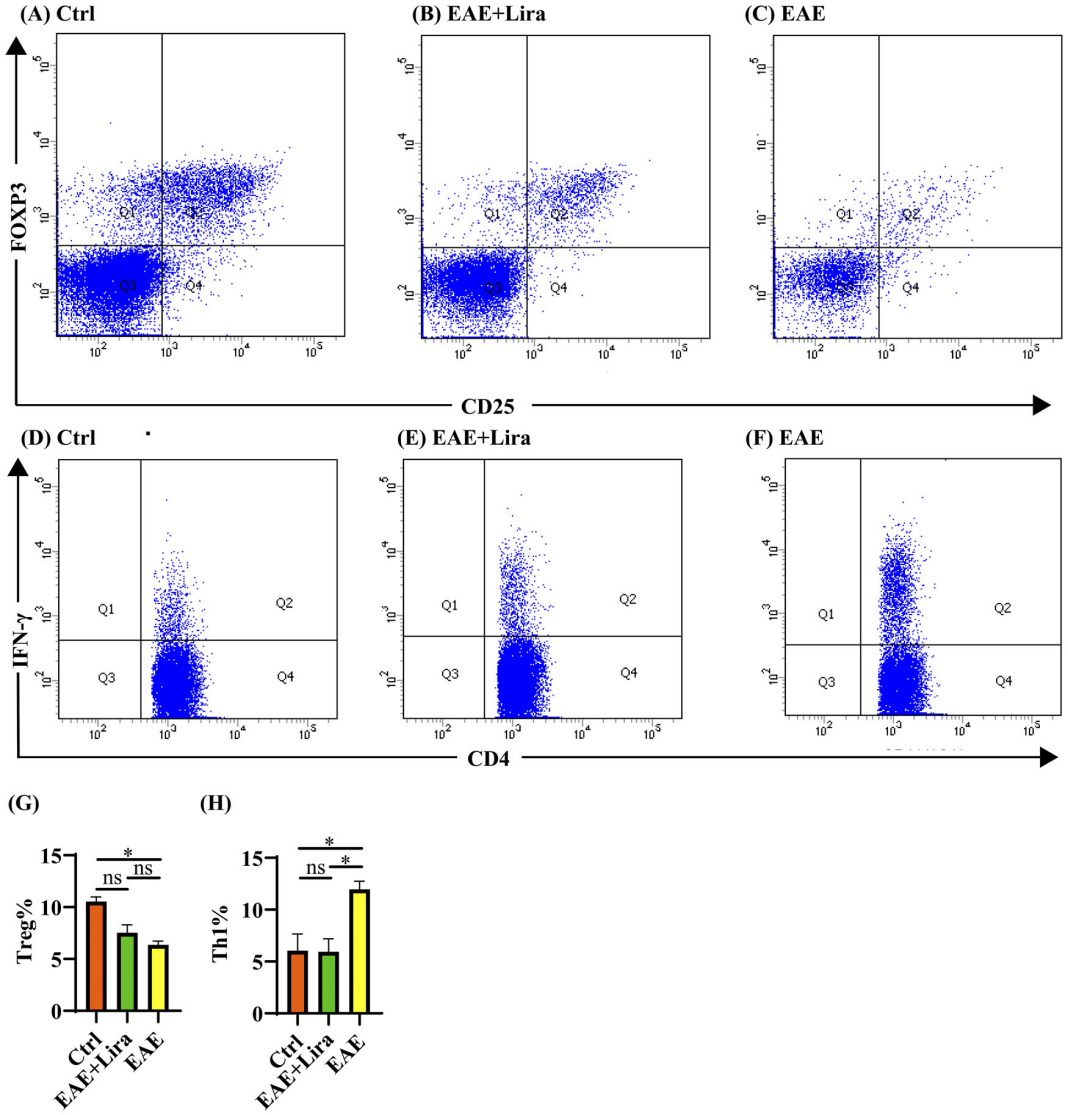
Flow cytometry of splenocytes. Data were shown with a format of mean ± SEM (n = 4 per group). (A–C) Representative flow cytometry results of CD4 + CD25 + Foxp3 + cells in splenocytes. The cells displayed in the figure have already been gated for CD4^+^, and the X‐axis shows the fluorescence intensity of CD25, while the Y‐axis shows the fluorescence intensity of Foxp3. (D–F) Representative flow cytometry results of CD4 + IFN‐γ + cells in splenocytes. The X‐axis shows the fluorescence intensity of CD4, while the Y‐axis shows the fluorescence intensity of IFN‐γ. (G, H) Quantitative statistics for the proportions of Treg and Th1 cells in splenocytes.

EAE induction significantly decreased the proportion of splenic Treg cells compared to controls (6.38% ± 0.72% versus 10.55% ± 0.87%, *p* = 0.013), but liraglutide treatment did not significantly elevate Treg proportions versus EAE alone (7.53% ± 1.54% versus 6.38% ± 0.72%, *p* = 0.975) (Figure 2A–C,G). Meanwhile, the proportion of Th1 cells significantly increased after MOG immunization (EAE group versus Ctrl group, 11.95% ± 1.58% versus 6.05% ± 3.23%, *p* = 0.025), and liraglutide injection significantly decreased the proportion to 5.94% ± 2.53% compared with the EAE group (*p* = 0.025) (Figure 2D–F,H).

## 4 Discussion

In this study, a neuroprotective role of liraglutide was observed in the EAE mouse model. The peak disease severity in EAE mice was attenuated, and the disease score curve flattened after liraglutide intervention. This finding aligns with our key finding that liraglutide significantly reduced the proportion of splenic Th1 cells, which constitute a critical proinflammatory subset. Specifically, while EAE induction led to a notable expansion of Th1 cells (characterized by IFN‐γ production), liraglutide administration mitigated this effect, lowering Th1 cell proportions to levels approaching those of healthy controls. The lack of change in Treg cells suggests that the protective effect of liraglutide in EAE may primarily stem from its ability to dampen Th1‐mediated neuroinflammation rather than enhance Treg‐mediated immunosuppression in splenocytes. These findings highlight a potential mechanism by which liraglutide modulates peripheral immune cell populations, particularly Th1 cells in the spleen, to reduce CNS inflammation in EAE. This is clinically relevant, as Th1 cells are known to drive the pathogenesis of MS (Liu et al. 2022, Angelini et al. 2023), and targeting their activation could offer a novel strategy to attenuate disease progression without broad immunosuppression.

The balance between Th1 and Treg cells is central to the immunopathogenesis of EAE and MS (Lu and Man 2025). In EAE, myelin antigen‐specific Th1 cells cross the blood‐brain barrier, secrete proinflammatory cytokines, and facilitate immune‐mediated damage to myelin and neurons (Karpus 2020). Conversely, Treg cells play a protective role by suppressing excessive Th1 and T helper 17 (Th17) responses, promoting immune tolerance, and limiting tissue injury in the CNS (Hu et al. 2024). Our results demonstrated that while EAE induction disrupted this balance by reducing Treg cell proportions (consistent with prior research) (Guo et al. 2021), liraglutide specifically countered the Th1 cell expansion without rescuing Treg cell numbers. This suggests that even in the absence of enhanced Treg cell function in splenocytes, dampening Th1 cell activation alone may be sufficient to alleviate disease severity in EAE. These observations are partially consistent with preclinical evidence linking GLP‐1RAs to anti‐inflammatory effects (Diz‐Chaves et al. 2024, Gharagozloo et al. 2023, Sadek et al. 2023, Ammar et al. 2022, Song et al. 2022, Gharagozloo et al. 2021, Kaye et al. 2024, Chiou et al. 2019), potentially positioning liraglutide as a candidate for modulating adaptive immunity in MS.

The mechanisms by which liraglutide regulates Th1 cells remain to be fully elucidated but likely involve interactions with GLP‐1Rs, which are expressed on various immune cells (Moschovaki Filippidou et al. 2020). Liraglutide may influence cell signaling pathways such as adenosine monophosphate‐activated protein kinase or nuclear factor‐κB (Diz‐Chaves et al. 2024, Ammar et al. 2022, Song et al. 2022), which are involved in T cell polarization and inflammation. However, whether this Th1‐specific regulation occurs directly via GLP1R on T cells or indirectly through crosstalk with other immune cell populations (e.g., antigen‐presenting cells) requires further investigation. Future studies exploring the molecular pathways underlying liraglutide's effects on Th1 cells, as well as its impact on CNS‐infiltrating immune cells, will be critical for translating these findings into potential therapeutic strategies for MS. Furthermore, emerging evidence has demonstrated that GLP‐1 modulates energy metabolism across multiple cell types (Zheng et al. 2021, Dasso et al. 2024, Qi et al. 2022), and that cellular energy metabolism plays a pivotal role in regulating Th1 cell differentiation (Peng et al. 2016, Rangel Rivera et al. 2021). Activated Th1 cells characteristically exhibit aerobic glycolysis as their predominant metabolic phenotype (Peng et al. 2016). Notably, hyperglycemia has been shown to disrupt Th1 differentiation through destabilization of signal transducer and activator of transcription 4 (Gray et al. 2024). Given its glucose‐modulating properties as a GLP‐1RA, investigating whether liraglutide influences splenic Th1 cell proportions in EAE through metabolic reprogramming presents a compelling mechanistic avenue for future research.

While our findings demonstrate that liraglutide modulates splenic Th1 cell responses in EAE, these observations may have important clinical implications for MS management. It would be unlikely that liraglutide intervention would serve more universally as a disease preventer, but may afford the clinician a tool to reduce establishment of chronic relapsing inflammation following a first clinical demyelinating event associated with MOG antibodies.

The study has several limitations that should be noted. First, although the anti‐demyelinating and anti‐inflammatory effects of liraglutide at the same dose in nerve tissues have been confirmed in previous research (Song et al. 2022), the pathological results of nerve tissues were not included in the current analysis. Second, the assessment of other immune cell populations within the spleen was not performed, which may have provided additional insights into the immune regulatory effects of liraglutide. Third, the absolute numbers of T cells were not measured, and immune cells in other lymphoid tissues, such as lymph nodes, were not evaluated, limiting the comprehensiveness of the immune profiling across different anatomical sites. Fourth, future studies should examine additional cytokines implicated in EAE pathogenesis, such as interleukin‐17, granulocyte‐macrophage colony‐stimulating factor, tumor necrosis factor‐α, and interleukin‐1β to more completely characterize liraglutide's immunomodulatory effects. Finally, the specific molecular and cellular mechanisms underlying the observed effects of liraglutide in EAE mice remain unclear, as the study did not investigate downstream signaling pathways or gene expression changes. Future research could address these gaps by utilizing advanced techniques such as transcriptome sequencing or single‐cell sequencing to explore the mechanistic pathways through which GLP‐1RAs modulate splenocyte function in the context of EAE.

## 5 Conclusion

The present preliminary findings demonstrate that liraglutide alleviates EAE severity probably through peripheral immunomodulatory effects of splenic T helper cells.

## Author Contributions

L. G. and B. L. were responsible for conceptualization and writing – review and editing. R. G. was responsible for funding acquisition, investigation and writing‐review & editing. Y. Z. was responsible for investigation and writing – review and editing. S. S. was responsible for funding acquisition, investigation, formal analysis, writing – original draft and writing – review and editing. Y. X. was responsible for formal analysis and writing – review and editing.

## Funding

This study was supported by the Medical Science Research Project of Hebei (No.20241859) and the Hebei Natural Science Foundation (No.H2023206107).

## Ethics Statement

All the experimental procedures were approved by the Experimental Ethics Committee of the Second Hospital of Hebei Medical University (2021‐AE043).

## Data Availability

The data that support the findings of this study are available from the corresponding author upon reasonable request.
